# Iodine in the Therapy of Graves' Disease: A Century After Henry S. Plummer

**DOI:** 10.1089/thy.2023.0068

**Published:** 2023-03-16

**Authors:** Peter A. Kopp

**Affiliations:** ^1^ Division of Endocrinology, Diabetes and Metabolism, University Hospital of Lausanne and University of Lausanne, Hôtel des Patients, Lausanne, Switzerland.; ^2^ Division of Endocrinology, Metabolism and Molecular Medicine, Feinberg School of Medicine, Northwestern University, Chicago, Illinois, USA.

In this issue of *Thyroid*, Uchida et al. present a mouse model of Graves' hyperthyroidism generated by immunization of BALB/c mice with the human thyrotropin receptor A-subunit.^1^ The investigators then compared the serum thyroxine (T4) and triiodothyronine (T3) levels, the intrathyroidal content of iodothyronines, as well as expression profiles of genes involved in thyroid hormonogenesis and secretion in untreated mice (GD-C) and mice treated with inorganic iodide (GD-NaI). Unimmunized BALB/c mice were used as controls. The results document a normalization of the serum T4 and T3 levels in the GD-NaI mice, whereas GD-C mice continued to have elevated serum concentrations relative to unimmunized mice.

Compared with unimmunized mice, GD-C mice had higher *intrathyroidal* concentrations of T3, reverse T3 (rT3), and T4. In the GD-NaI mice, the mean intrathyroidal T4 and rT3 concentrations were roughly twofold higher in comparison with the GD-C mice. Transcriptome analyses showed an upregulation of genes coding for proteins involved in thyroid hormone synthesis, transport processes, and redox balance in the GD-C mice. In contrast, treatment with iodine inhibited the upregulation of these genes in the GD-NaI mice. More specifically, GD-C mice showed an upregulation of key genes such as *Tshr*, *Tpo*, *Dio1*, and the thyroid hormone transporter *Slco4a1* compared with controls. In GD-NaI mice, genes such as *Slc5a5 (Nis)*, *Slc26a4 (pendrin)*, *Tpo*, *Duox2* and *Duoxa2*, *Dio1*, and *Slco4a1* were downregulated, whereas *Dio3* was upregulated.

The data document that treatment with inorganic iodide results (1) in a decrease in thyroid hormone synthesis, (2) a decrease in thyroid hormone secretion, (3) an intrathyroidal increase of T4 and rT3, and (4) a normalization of serum T3 and T4. In aggregate, these studies add additional mechanistic details on the effects of inorganic iodide in Graves' disease,^1^ a treatment that has been introduced a century ago by Henry S. Plummer (1874–1936; see Fig. 1, also cover figure of this issue).^2–5^ This latter aspect is not mentioned by Uchida et al., and it is relevant to briefly summarize the history of the use of iodine in the treatment of goiter and hyperthyroidism.

**FIG. 1.**
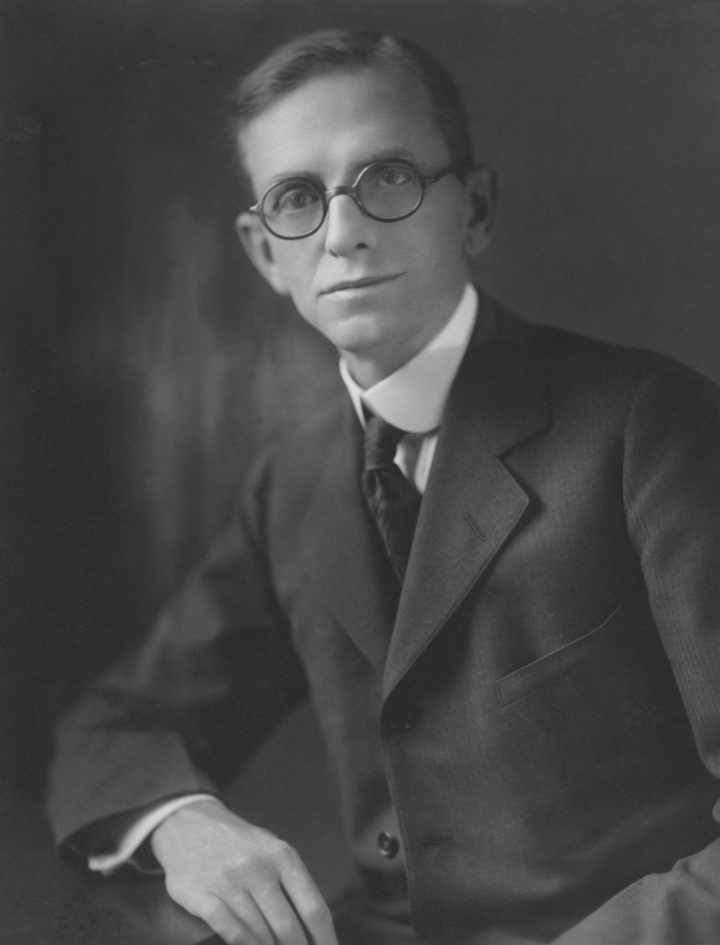
Dr. Henry Stanley Plummer (1874–1936), past president of the American Thyroid Association in 1933.

After its discovery in 1811 by Chatin, iodine was used in the therapy of numerous diseases. Coindet, practicing in Geneva, Switzerland, used potassium iodide for the therapy of goiter.^6^ In 1821, he reported that this often led to remarkable volume reductions but that some patients developed major toxic side effects, including severe tachycardia.^7^ Without understanding the underlying mechanism, Coindet was confronted with iodine-induced thyrotoxicosis. Switzerland was severely iodine deficient at that time and, hence, many of these patients had goiters with autonomously functioning thyroid nodules. In 1910, Kocher coined the term *Jod-Basedow* (Jod = iodine in German) to describe this form of hyperthyroidism.^8^ Because of the risk of inducing thyrotoxicosis in goitrous patients, the use of iodine then fell into disrepute.

But with time it also became apparent that there are two distinct etiologies of the syndrome that we now call hyperthyroidism. Some patients present with *simple or adenomatous goiter*, others with *exophthalmic goiter.*^5,9^ *Exophthalmic goiter* had been recognized independently by several individuals, including Caleb Parry (1786), Giuseppe Flajani (1833), Robert Graves (1840), and Carl Adolph von Basedow (1840).^10,11^ In 1909, William Osler formulated the hypothesis that *exophthalmic goiter* (now Graves' disease in the English literature) is “due to disturbed function of the thyroid gland, probably a hypersecretion of certain materials which induce a sort of chronic toxaemia.”^10^ The treatment consisted in thyroidectomy, which was generally associated with a high mortality in the hands of most surgeons.

It was also believed that Graves' disease was a form of “dysthyroidism” in which the gland secretes an abnormal thyroid hormone or toxin. Plummer, practicing at the Mayo clinic, reasoned that the secreted product could perhaps be iodine deficient. Henry Plummer joined the Mayo clinic in 1901, became the fourth Mayo partner, had a major impact on the development and structure of the burgeoning firm, and made numerous contributions to medicine.^12,13^ In 1922, Plummer administered iodine in the form of Lugol's solution (an aqueous solution containing potassium iodide and iodine) to patients with exophthalmic goiter scheduled for thyroidectomy. Surprisingly, the signs and symptoms of hyperthyroidism often disappeared within a few days.^2–5^

Equally important, the surgical mortality dropped from about 4–5% at the Mayo Clinic, an expert center, to <1%. In addition to the impact on thyroidal blood flow, it is likely that the decrease or normalization of the peripheral thyroid hormone levels contributed to the improvements in outcomes. This so-called *Plummer effect* describes the fact that large intrathyroidal concentrations of iodide inhibit hormone secretion in patients with Graves' disease.^14^ Remarkably, Armand Trousseau documented the following observation already in 1862: “However, …, it happens, …, that iodine preparations can be tolerated without damage and even with a semblance of improvement by certain persons suffering from Graves' disease.”*^,15^ Starr et al. at the Massachusetts General Hospital confirmed the findings of the Mayo group,^16^ and many subsequent clinical studies further corroborated the validity of administering iodine to patients with Graves' disease.^17–20^

Plummer's name lives on in the Plummer–Vinson syndrome (sideropenic dysphagia) and, relevant to thyroidologists, in Plummer's disease, which designates hyperthyroidism due to one or several autonomous thyroid adenomas. Of note, Plummer was the 9th president of the American Association for the Study of Goiter, now the American Thyroid Association, in 1933.^21^

The chronic inhibitory effect of inorganic iodide in patients with Graves' disease needs to be distinguished from the well-known autoregulatory *Wolff-Chaikoff effect*, which describes the transient inhibitory effect of high concentrations of iodide on *iodine organification* in the thyroid *in vivo.*^22,23^ The seminal study by Wolff and Chaikoff was published in 1948 and had been preceded by the demonstration of an acute inhibitory effect of iodide *in vitro* in 1944.^24^

To this date, the administration of iodine is one of the cornerstones in the treatment of thyroid storm,^25,26^ and it can also be used as an alternative to thionamides in selected instances.^27–29^

While certain details may be different in the human thyroid (e.g., the type of involved thyroid hormone transporter(s)), the study by Uchida et al. discussed earlier adds further detail on the role of inorganic iodide in regulating thyroid cell function—a century after the observations of Plummer, and two centuries after the reports of Coindet.

## Notes

^*^
Original text in French: “Cependant, …, il arrive, …, que les préparations iodées, peuvent être supportées sans dommage et même avec un semblant d'amélioration par certaines personnes atteintes de la maladie de Graves.”

## References

[1] Uchida T, Shimamura M, Taka H, et al. The effect of long-term inorganic iodine on intrathyroidal iodothyronine content and gene expression in mice with Graves' hyperthyroidism. Thyroid 2023;33(3):330–337. 10.1089/thy.2022.0496 PMC10024588 36565031

[2] Plummer H. Results of administering iodin to patients having exophthalmic goiter. J Am Med Assoc 1923;80(26):1955–1956

[3] Plummer H, Boothby W. The value of iodin in exophthalmic goiter. Illinois Med J 1924 xlvi(6):401–407.

[4] Plummer H, Boothby W. The value of iodin in exophthalmic goiter. J Iowa State Med Soc 1924;xiv(2):66–73.

[5] Plummer H. The results of iodin administration in exophthalmic goiter. Trans Am Surg Assoc 1926;42:541–556.

[6] Coindet J. Découverte d'un nouveau remède contre le goitre. [Discovery of a new cure for goiter. Article in French]. Bibliothèque Universelle, Sciences et Arts, Genève 1820;14:190–198.

[7] Coindet J. Nouvelles études sur l'iode et sur les précautions à prendre dans le traitement du goitre par ce nouveau remède. [New studies on iodine and the precautions to be taken in the treatment of goiter with this new drug. Article in French]. Bibliothèque universelle, Sciences et Arts, Genève 1821;16:140–152.

[8] Kocher T. Über Jodbasedow. [On iodine-Basedow. Article in German]. Langenbecks Arch Klin Chir 1910;92:1166–1193.

[9] Plummer H. The clinical and pathological relationship of simple and exophthalmic goiter. Am J Med Sci 1913;146:790–795.

[10] Ahmed AM, Ahmed NH. History of disorders of thyroid dysfunction. sEast Mediterr Health J 2005;11(3):459–469. 16602467

[11] Lindholm J, Laurberg P. Hyperthyroidism, exophthalmos, and goiter: Historical notes on the orbitopathy. Thyroid 2010;20(3):291–300. 20187784 10.1089/thy.2009.0340

[12] Sawin C. Henry S. Plummer (1874–1936), iodine for hyperthyroidism, and Plummer's disease. Endocrinologist 2003;13(3):149–152.

[13] Nelson C. Dr. Plummer: The Fourth Mayo Partner. Mayo Roots—Profiling the Origins of the Mayo Clinic. Mayo Foundation: Rochester, MN; 1990; pp. 129–130.

[14] Saller B, Fink H, Mann K. Kinetics of acute and chronic iodine excess. Exp Clin Endocrinol Diabetes 1998;106 Suppl 3:S34–S38. 10.1055/s-0029-1212044 9865552

[15] Trousseau A. Du goitre exophthalmique, ou maladie de Graves. [On exophthalmic goiter, or Graves' disease. Article in French]. Clinique médicale de l'Hôtel-Dieu de Paris. Tome 2. Librairie J.-B. Baillère et Fils: Paris; 1885; pp. 551–600.

[16] Starr P, Walcott H, Segall H, et al. The effect of iodin in exophthalmic goiter. Arch Intern Med (Chic) 1924;34(3):355–364.

[17] Jackson AS. The effect of the administration of iodine upon exophthalmic goitre: A study of seventy cases thus treated. Ann Surg 1925;81(4):739–747. 17865232 10.1097/00000658-192504000-00002 PMC1399985

[18] Goldsmith RE, Eisele ML. The effect of iodide on the release of thyroid hormone in hyperthyroidism. J Clin Endocrinol Metab 1956;16(1):130–137. 13278387 10.1210/jcem-16-1-130

[19] Nagataki S, Shizume K, Nakao K. Effect of iodide on thyroidal iodine turnover in hyperthyroid subjects. J Clin Endocrinol Metab 1970;30(4):469–478. 5435286 10.1210/jcem-30-4-469

[20] Wartofsky L, Ransil BJ, Ingbar SH. Inhibition by iodine of the release of thyroxine from the thyroid glands of patients with thyrotoxicosis. J Clin Invest 1970;49(1):78–86. 5409810 10.1172/JCI106225 PMC322446

[21] American Thyroid Association. Past Presidents 2023. Available from: https://www.thyroid.org/members-only/member-resources/leadership/past-presidents/ [Last accessed: March 5, 2023].

[22] Wolff J, Chaikoff IL. Plasma inorganic iodide as a homeostatic regulator of thyroid function. J Biol Chem 1948;174(2):555–564. 18865621

[23] Wolff J, Chaikoff IL. The inhibitory action of iodide upon organic binding of iodine by the normal thyroid gland. J Biol Chem 1948;172(2):855. 18901214

[24] Morton M, Chaikoff I, Rosenfeld S. Inhibiting effect of inorganic iodide on the formation in vitro of thyroxine and diiodotyrosine by surviving thyroid tissue. J Biol Chem 1944;154:381–387.

[25] Ross DS, Burch HB, Cooper DS, et al. 2016 American Thyroid Association guidelines for diagnosis and management of hyperthyroidism and other causes of thyrotoxicosis. Thyroid 2016;26(10):1343–1421. 27521067 10.1089/thy.2016.0229

[26] Feldt-Rasmussen U, Emerson CH, Ross DS, et al. Thoughts on the Japanese and American perspectives on thyroid storm. Thyroid 2019;29(8):1033–1035. 31140377 10.1089/thy.2019.0010

[27] Okamura K, Sato K, Fujikawa M, et al. Remission after potassium iodide therapy in patients with Graves' hyperthyroidism exhibiting thionamide-associated side effects. J Clin Endocrinol Metab 2014;99(11):3995–4002. 25144628 10.1210/jc.2013-4466

[28] Uchida T, Goto H, Kasai T, et al. Therapeutic effectiveness of potassium iodine in drug-naive patients with Graves' disease: A single-center experience. Endocrine 2014;47(2):506–511. 24493028 10.1007/s12020-014-0171-8

[29] Yoshihara A, Noh JY, Watanabe N, et al. Substituting potassium iodide for methimazole as the treatment for Graves' disease during the first trimester may reduce the incidence of congenital anomalies: A retrospective study at a single medical institution in Japan. Thyroid 2015;25(10):1155–1161. 26222916 10.1089/thy.2014.0581

